# Protocol to identify E3 ligases amenable to biodegraders using a cell-based screening

**DOI:** 10.1016/j.xpro.2024.103413

**Published:** 2024-10-24

**Authors:** Marie Sorbara, Margot Cristol, Anaïs Cornebois, Klervi Desrumeaux, Pierre Cordelier, Nicolas Bery

**Affiliations:** 1 Université de Toulouse, Inserm, CNRS, Université Toulouse III-Paul Sabatier, Centre de Recherches en Cancérologie de Toulouse, 31100 Toulouse, France; 2 Sanofi, Large Molecule Research, 94400 Vitry-sur-Seine, France

**Keywords:** cell biology, cell culture, cell-based assays, molecular biology, antibody

## Abstract

Here, we provide a protocol for the identification of E3 ubiquitin ligases that are functional when implemented as biodegraders using a cell-based screening assay. We describe steps for establishing a stable cell line expressing a GFP-tagged protein of interest (POI), preparing a sub-library of E3 ligases to screen, and performing the cell-based screening. This protocol can be broadly applied to identify any functional E3 ligase in a biodegrader setting.

For complete details on the use and execution of this protocol, please refer to Cornebois et al.^1^

## Highlights

• Production of a stable cell line expressing a GFP-tagged protein of interest (POI)
• Preparation of a homogenous population of cells expressing the GFP-tagged POI
• Selection and design of the E3 ligase to screen and cloning in the biodegrader vector
• Cell-based screening of E3 ligases amenable to biodegraders

## Abstract (editor highlights)

Publisher’s note: Undertaking any experimental protocol requires adherence to local institutional guidelines for laboratory safety and ethics.

## Graphical abstract

**Figure:**
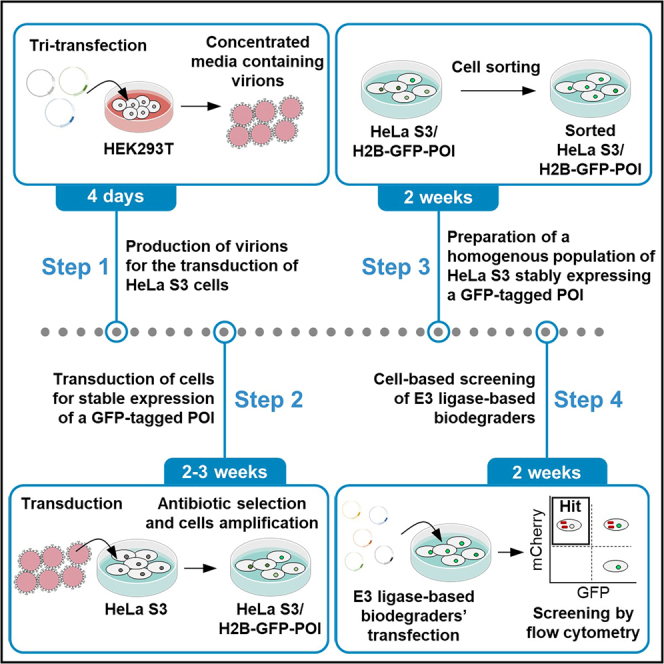

## Before you begin

This protocol was used in a publication to identify E3 ubiquitin ligases that function as biodegrader or bioPROTAC.^1^ Biodegraders are a fusion between an E3 ligase and an intracellular POI-specific protein binder. We have implemented a cell-based screening that enables the direct monitoring of fluorescent-tagged POI degradation by functional biodegraders. A stable cell line with homogenous expression of the GFP-tagged POI needs to be prepared before starting the screening. Indeed, as previously indicated,^2^ the establishment of such stable cell lines is mandatory for the success of the screening. The screening was performed by flow cytometry, which allows a quantitative measurement of the POI degradation. The POI (here GFP-ALFA-KRAS^G12V^_166_) was fused to the histone H2B for a chromatin localization. Any fluorescent-tagged POI fused to any subcellular targeting sequence could be used as long as an intracellular protein binder targeting this POI is available. In this protocol, we employ intracellular single domain antibody (sdAb)^3^ as binder of the POI but other protein binder formats could be used such as DARPins,^4^ monobodies^5^ or affimers.^6^ In addition, this protocol could be applied to select intracellular sdAbs that deplete the POI when fused to a known E3 ligase.

### Experimental considerations

**Figure 1:**
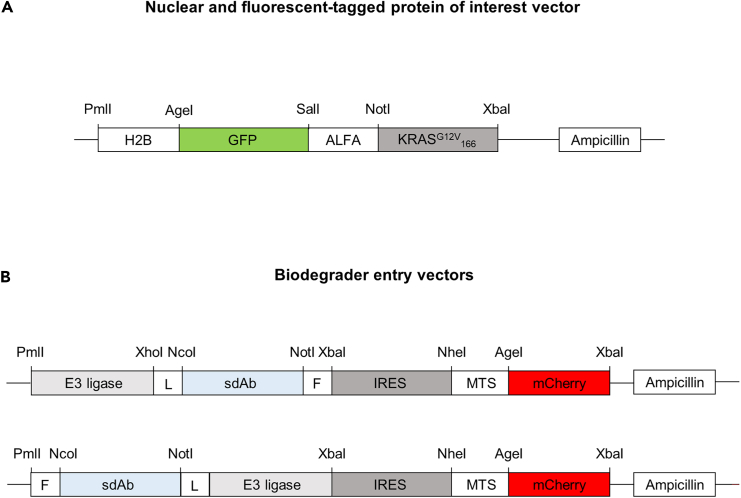
Schematic diagrams of the vectors used in this protocol (A) H2B-GFP-ALFA-KRAS^G12V^_166_ is the fluorescent protein of interest (POI) entry vector to express the POI (GFP-ALFA-KRAS^G12V^_166_) in fusion to the histone H2B. Any POI can be inserted in this vector using AgeI/XbaI restriction sites. The different restriction sites used to construct these vectors are shown on the diagrams. (B) E3 ligase-sdAb or sdAb-E3 ligase are the biodegrader entry vectors. NcoI/NotI are the restriction sites used to insert any antibody of interest in fusion with the E3 ligase either on its amino terminal (top vector) or carboxy terminal end (bottom vector). sdAb, single domain antibody; MTS, mitochondrial targeting sequence; F, FLAG tag; L, linker.

In this protocol, three entry vectors are used and were described elsewhere^1^: one for the chimeric POI (Figure 1A) and two for the biodegraders (Figure 1B). Here, the POI is GFP-ALFA-KRAS^G12V^_166_ and an intracellular protein binder is available for each component of this POI. GFP is used as fluorescent reporter to quantify the degradation of the chimeric POI by flow cytometry. It could be replaced by any fluorescent protein detectable by flow cytometry (between AgeI/SalI restriction sites). The ALFA tag is a small size tag (13 amino acids), for which a high affinity sdAb is available (26 pM, named sdAb ALFA).^7^ KRAS^G12V^_166_ is a G12V mutant of KRAS deleted of its 22 last carboxyterminal amino acids that does not bind to the plasma membrane, for which multiple intracellular protein binders are available.^8^^,^^9^^,^^10^^,^^11^ The ALFA tag and KRAS can be removed using the restriction sites indicated on Figure 1A. The POI is further fused to the histone H2B, which will localize the POI to the chromatin. The targeting localization sequence can be changed by using the restriction sites PmlI/AgeI.

The two other vectors employed in this protocol are the biodegraders entry vector (Figure 1B). These vectors express an intracellular sdAb fused to an E3 ligase either on its amino terminal end or on its carboxy terminal end. A glycine/serine linker (L) is inserted between the sdAb and the E3 ligase. A FLAG tag (F) is on each construct to enable the detection of the biodegraders by Western blot for instance. IRES-MTS-mCherry is used as fluorescent reporter to visualize transfected cells. MTS is a mitochondrial targeting sequence from subunit VIII of human cytochrome c oxidase fused to a mCherry (red fluorescent protein). The sdAb and E3 ligase can be replaced by using the indicated restriction sites (Figure 1B).

## Key resources table

**Table:** 

REAGENT or RESOURCE	SOURCE	IDENTIFIER
**Antibodies**		

FLAG mouse antibody (1/2,500 dilution)	Sigma	Cat#F1804; RRID: AB_262044
α-tubulin rabbit antibody (1/2,000 dilution)	Abcam	Cat#ab4074; RRID: AB_2288001

**Chemicals, peptides, and recombinant proteins**		

Polybrene	Sigma	Cat#107689
Blasticidin	InvivoGen	Cat#ant-bl-05
Trypsin-EDTA solution	Sigma	Cat#T3924-100ML
Penicillin-streptomycin (10,000 U/mL)	Gibco	Cat#15140-122
DMEM	Sigma	Cat#D6429-500ML
Fetal bovine serum	Gibco	Cat#A5256701
Epoxomicin	Sigma	Cat#E3652-50UG
Dimethyl sulfoxide	Sigma	Cat#D8418

**Critical commercial assays**		

jetPRIME	Polyplus Transfection	Cat#101000046
NucleoBond Xtra Maxi EF	MACHEREY-NAGEL	Cat#740424.50

**Experimental models: Cell lines**		

HEK293T	ATCC	Cat#CRL-3216; RRID: CVCL_0063
HeLa S3	ATCC	Cat#CCL-2.2; RRID: CVCL_0058

**Recombinant DNA**		

pLenti-H2B-GFP-ALFA-KRAS^G12V^_166_	Cornebois et al.^1^	N/A
psPAX2	Addgene	Cat#12260
pMD2.G	Addgene	Cat#12259
pEF-E3 ligase-Linker-sdAb-FLAG-IRES-MTS-mCherry	Cornebois et al.^1^	N/A
pEF-FLAG-sdAb-Linker-E3 ligase-IRES-MTS-mCherry	Cornebois et al.^1^	N/A

**Software and algorithms**		

FlowJo 10	FlowJo software	N/A

**Other**		

MACSQuant VYB flow cytometer	Miltenyi	N/A
FACSMelody	BD Biosciences	N/A
0.45 μm PES filter	Sigma	Cat#SLHPR33RS
0.2 μm filter	ClearLine	Cat#146560
Vivaspin 20 PES 100 kDa MWCO	Sartorius	Cat#VS2042
12-well plates	Falcon	Cat#353043
6-well plates	Falcon	Cat#353046
T25 flasks	Falcon	Cat#353109
T75 flasks	Falcon	Cat#353136
T175 flasks	Thermo Scientific	Cat#159910
100 mm dishes	Falcon	Cat#353003
15 mL tubes	Falcon	Cat#352096
5 mL round-bottom tube	Falcon	Cat#352052

## Materials and equipment

### Cell culture

Here we specify the reagents used for the cell culture of HEK293T and HeLa S3 cells (wild-type and stable cells).

***Note:*** Mycoplasma free cell culture is performed in a sterile tissue culture hood under aseptic conditions and cells are maintained in a humidified 37°C incubator with 5% CO_2_.

HEK293T cell medium: DMEM supplemented with 10% Fetal Bovine Serum (FBS) and 100 U/mL Penicillin/Streptomycin.

HeLa S3 cell medium: DMEM supplemented with 10% FBS and 1% Penicillin/Streptomycin with or without 3 μg mL^–1^ of Blasticidin.

### Polybrene stock solution preparation

Dissolve polybrene at a concentration of 8 mg mL^–1^ in ddH_2_O and sterilize the solution by passing it through a 0.2 μm filter.

**CRITICAL:** Store the polybrene solution as small aliquots (50 μL) at −20°C (stable up to 2 years). Discard after use.

### Epoxomicin stock solution preparation

Resuspend epoxomicin at a concentration of 1 mM in dimethyl sulfoxide (DMSO) in a sterile tissue culture hood under aseptic conditions.

**CRITICAL:** Store the epoxomicin solution as small aliquots (10 μL) at −20°C (stable up to 2 years). Discard after use.

### Equipment for the cell sorting

Fluorescence-activated Cell Sorting (FACS) MELODY (BD Biosciences).

***Alternatives:*** Any FACS instrument that has a 488 nm (GFP) laser can be used for the cell sorting.

### Equipment for the screen

Flow cytometer: MACSQuant VYB (Miltenyi).

***Alternatives:*** Any flow cytometer that has a 488 nm (GFP) and a 561 nm (mCherry) laser can be used for the screening.

## Step-by-step method details

### Production of virions for the transduction of HeLa S3 cells

**Timing: 4 days**

Before transducing target cells, the lentiviral vectors have to be produced in HEK293T cells by performing a tri-transfection with plasmids coding for the VSV-G envelop (pMD2.G), the packaging (psPAX2) and the transgene of interest. This protocol is for the transduction of one cell line with one lentiviral vector. It can be scaled up depending on the number of cell lines and/or of lentiviral vector to transduce.

1. Plate 4.5 × 10^6^ HEK293T cells in one 100 mm dish in 9.5 mL of supplemented DMEM.
2. 24 h later, transfect the cells with the JetPRIME reagent.
  a. Add the following plasmids: 1.5 μg of pMD2.G, 4 μg of psPAX2 and 6 μg of pLenti-H2B-GFP-ALFA-KRAS^G12V^_166_ (or any lentivector of choice) to 477 μL of JetPRIME buffer.
  b. Vortex 10 s and centrifuge 3 s at 2,000 × *g*.
  c. Add 23 μL of JetPRIME reagent, vortex 10 s and centrifuge 3 s at 2,000 × *g*.
  d. Incubate at 22°C for 10 min without agitation.
  e. Add the mix drop by drop on the cells.

**CRITICAL:** Biosafety precautions: proper handling of lentiviral vector should be followed as outlined by your institution's Environmental Health and Safety Office.

***Note:*** The plasmids used for the tri-transfection step should be extracted and purified from *E.coli* cultures using an endotoxin free purification kit following the manufacturer protocol (e.g. NucleoBond Xtra Maxi EF).

3. Collect the virions-containing media in a 50 mL Falcon tube 48 h after transfection. Troubleshooting 1.
4. Centrifuge 5 min at 850 × *g* at 22°C to remove remaining HEK293T cells and cells debris.
5. Pass the supernatant through a 0.45 μm filter.
6. Add the filtered media in a Vivaspin concentrator (100 kDa cutoff).
7. Centrifuge for 30 min at 1,300 × *g* at 22°C. Repeat the centrifugation step until the remaining volume is around 0.5 mL.
8. Collect the 0.5 mL of concentrated virions-containing media in a 1.5 mL Eppendorf tube.

### Transduction of HeLa S3 cells for stable expression of a GFP-tagged POI

**Timing: 2–3 weeks**

Transduction of HeLa S3 with a GFP-tagged protein of interest (POI, here H2B-GFP-ALFA-KRAS^G12V^_166_) lentivector will allow the selection of cells that stably express a green fluorescent antigen localized to the chromatin. This will make the screen easier and more reliable than transient transfection of the antigen. The POI is localized to the chromatin through histone H2B but it could be targeted to any subcellular localization of choice by adding a targeting localization sequence (e.g., nuclear exclusion sequence, plasma membrane sequence, …) or not targeted (diffuse localization).

9. 24 h after transfection (step 2), plate 110,000 HeLa S3 cells per well of a 6-well plate in 2 mL of supplemented DMEM.

***Note:*** Usually, three wells of a 6 well plate are seeded with the target cells for transduction by one lentiviral vector the following day.

10. Aspirate the media from HeLa S3 cells seeded in the 6 well plate.
11. Add 1 mL of supplemented DMEM containing 8 μg mL^–1^ of polybrene.

***Note:*** Polybrene is a positively charged polymer that enhances lentiviral vector transduction efficiency by facilitating viral entry. In our hands, 8 μg.mL^–1^ of polybrene works fine with all cell lines we tested.

12. Add 150 μL of concentrated virions-containing media directly on each well of the 6 well plate and incubate at 37°C, 5% CO_2_.

***Note:*** Virions-containing media should be used fresh for optimal transduction.

**Pause point:** Otherwise, the virion-containing media can be aliquoted and stored at −80°C, which will greatly decrease the transduction efficiency.

13. After 36 h of incubation (Troubleshooting 2), pass the cells.
  a. Aspirate the media.
  b. Wash with 1 mL of 1X PBS and aspirate the PBS.
  c. Add 500 μL of trypsin-EDTA solution.
  d. Incubate 3 min at 37°C, 5% CO_2_ until the cells detached.
  e. Prepare a 25 cm^2^ flask (T-25) with 5 mL of supplemented DMEM.
  f. Once detached, pipet the 500 μL of trypsinized cells and add it in the 25 cm^2^ flask.
14. Change the medium 24 h later by adding 5 mL of supplemented DMEM containing 3 μg mL^–1^ of blasticidin for antibiotic selection.
15. Amplify the cells by increasing the surface of culture every time they are confluent and by keeping the blasticidin for antibiotic selection (Troubleshooting 3).
  a. Amplify the cells from a T-25 to a T-75.
  b. Amplify the cells then from a T-75 to a T-175.
  c. Freeze 5–6 aliquots (5 × 10^6^ cells per vial) as stock.
  d. Store them in liquid nitrogen.

***Note:*** The amplification step should take around two weeks.

### Preparation of a homogenous population of HeLa S3 stably expressing a GFP-tagged POI

**Timing: 2 weeks**

**Figure 2:**
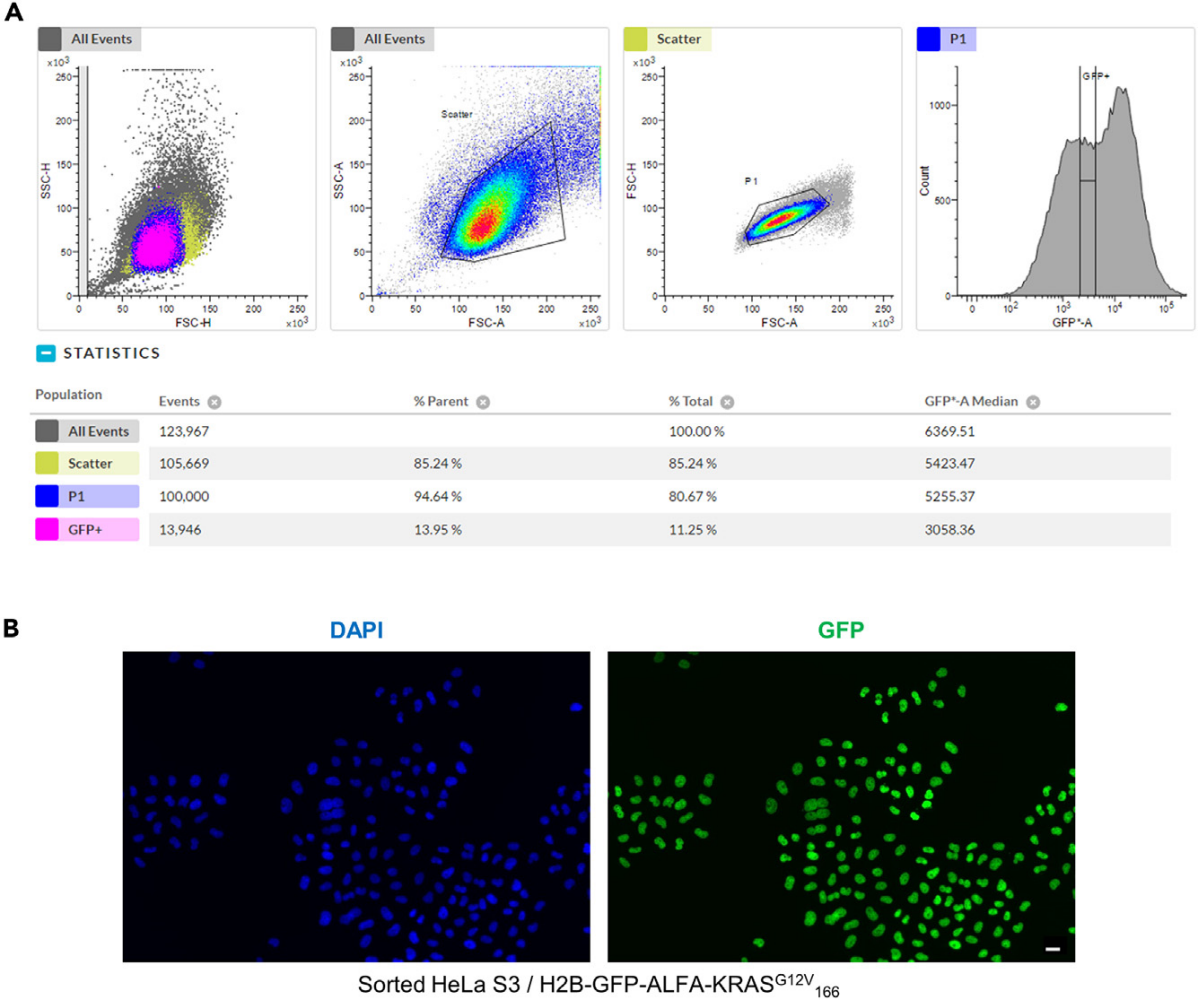
Example of cell sorting for HeLa S3 / H2B-GFP-ALFA-KRAS^G12V^_166_ (A) HeLa S3 cells stably expressing H2B-GFP-ALFA-KRAS^G12V^_166_ are sorted by a FACS MELODY to get a homogenous cell population expressing the GFP. (B) Representative image of the cells after cell sorting. DAPI is used to stain the DNA (blue channel) and GFP is directly visualized on the green channel. Scale bar, 10 μm.

After amplification of the transduced HeLa S3 stably expressing the GFP-POI and before using them for the screening, it is strongly advised to sort the cells in order to obtain a homogenous population of cells regarding their GFP fluorescence (Figures 2A and 2B). A homogenous population will enable more accurate and reliable results from the screening, especially by reducing false positive hits.

16. Detached HeLa S3 cells stably expressing H2B-GFP-ALFA-KRAS^G12V^_166_.
  a. Add 2.5 mL of trypsin-EDTA solution into a T-175 at 70%–80% confluence.
  b. Add 8.5 mL of supplemented DMEM to neutralize the trypsin.
  c. Mix gently by pipetting up and down.
17. Count the cells with a hemocytometer.
18. Dilute cells at 10 × 10^6^ cells / mL in PBS-4% FBS.
19. Sort the cells using a narrow gate on the GFP positive cell population (see Figure 2A) with the FACS MELODY placed in a sterile tissue culture hood under aseptic conditions.
  a. Collect the cells in a 15 mL Falcon tube containing 2 mL of supplemented DMEM.
  b. Sort around 0.5–1 × 10^6^ cells.
    **CRITICAL:** Cells should be sorted under aseptic conditions to avoid any contamination while they regrow.
  c. Centrifuge the 15 mL Falcon tube 5 min at 800 × *g* at 22°C.
  d. Aspirate the supernatant.
  e. Add 5 mL of supplemented DMEM and gently resuspend the cells.
  f. Add the cells in a T-25 flask and incubate at 37°C, 5% CO_2_.
  g. Amplify the cells and freeze a stock (at least 8–10 vials with 5 × 10^6^ cells / vial).

### Cell-based screening of E3 ligase-based biodegraders

**Timing: 2–3 weeks**

Before performing the screening, it is important to select and design the E3 ligases that will be screened. There are more than 600 E3 ligases in the human genome divided in three main families: Really Interesting New gene (RING), Homologous to E6-AP carboxyl terminus (HECT) and RING between RING (RBR) E3s.^12^ We chose to pick E3 ligases (i) from different families (ii) with ubiquitous expression across tissues and (iii) without any specific subcellular localization by using the UbiHub online resource.^13^ This selection process can be modified depending on the features that are required for the E3 ligase. Here, we prioritized versatile E3 ligases, i.e., E3s that are functional in as many settings as possible. Once selected, we designed the truncation of the E3s by replacing the natural substrate recognition motif of each E3 ligase receptor with the sdAb ALFA protein binder. This identification of the natural substrate motif is made via an analysis of the E3 domains using Uniprot online protein database (www.uniprot.org). It will decide on the position of the E3 ligase on the sdAb. If no information is available, then use the full-length E3 ligase. Next, the E3 ligases were synthesized by gene synthesis including two restriction sites: PmlI/XhoI or NotI/XbaI for Nter or Cter fusion on the sdAb, respectively (see Figure 1B).

**Figure 3:**
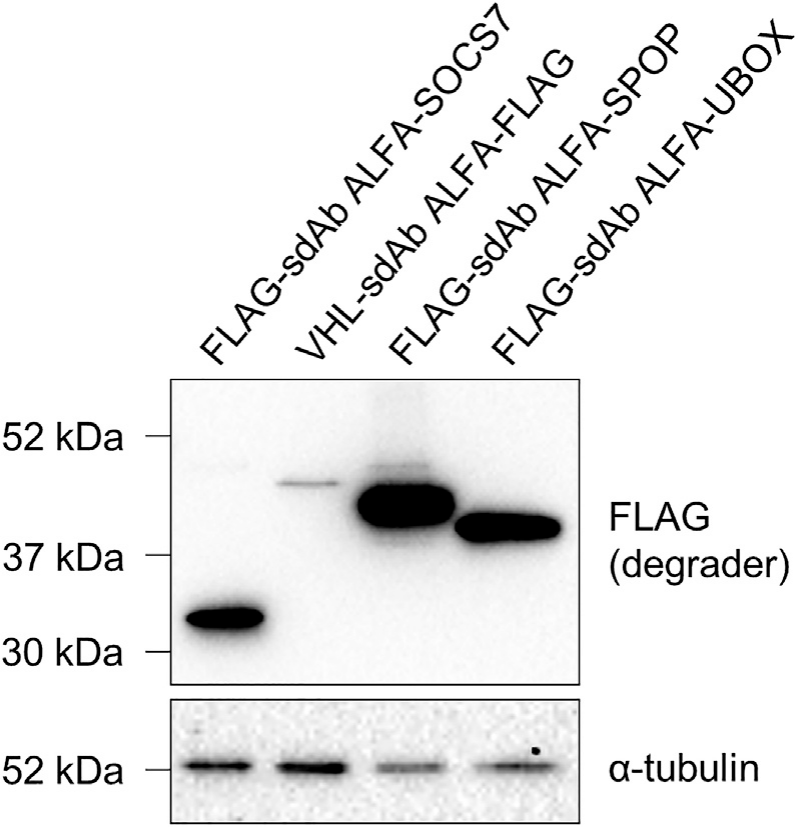
Example of Western blot to control the level of expression of the biodegraders The control of expression of the indicated biodegraders (via FLAG tag) was analyzed by Western blot. α-tubulin is the loading control.

**CRITICAL:** Check the expression level of all the biodegraders by Western blot before performing the screening (if feasible with the number of biodegraders to screen) as performed in Cornebois et al.^1^ (see an example in Figure 3).

**Figure 4:**
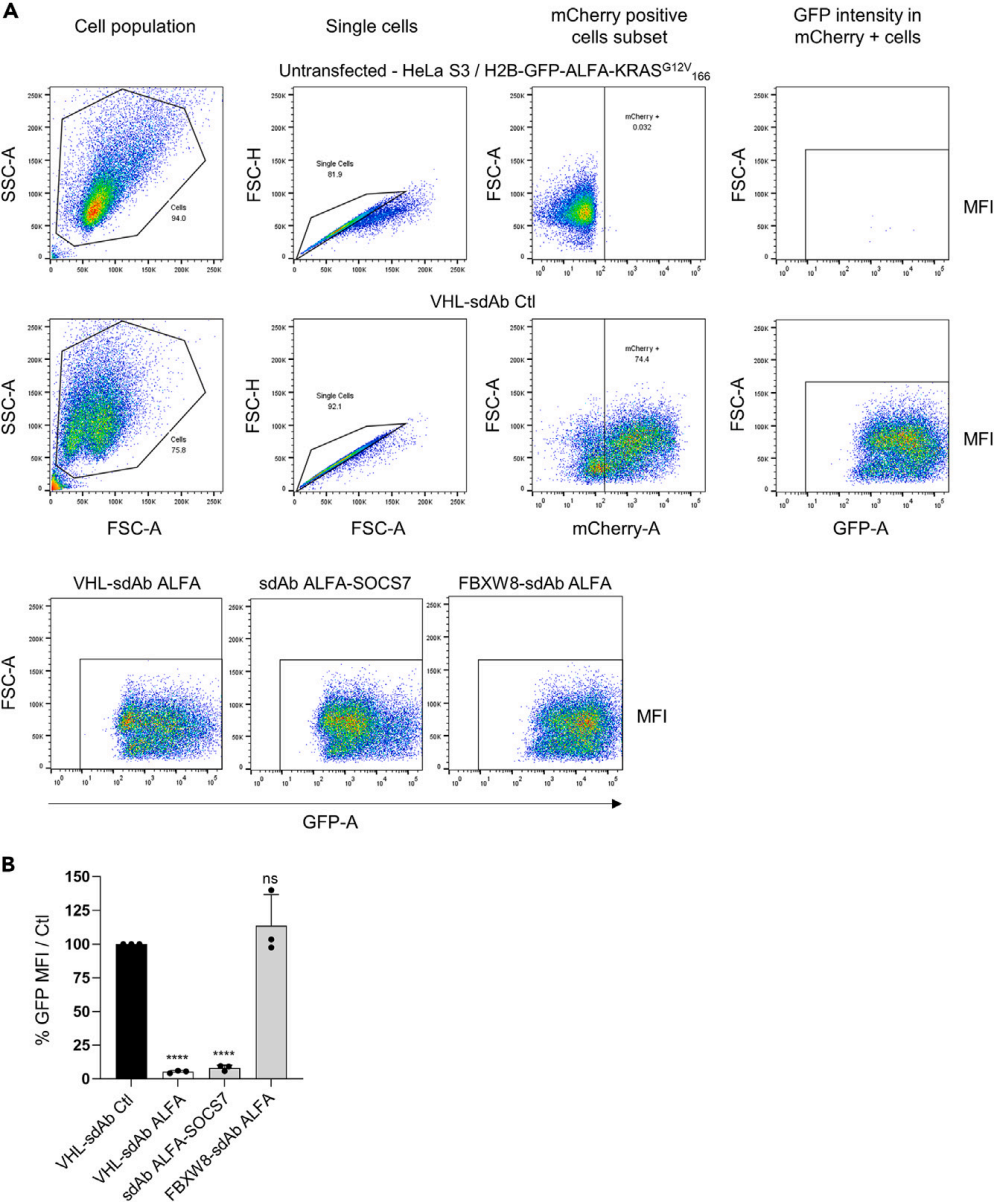
Expected results from the cell-based screening of E3 ligases amenable to biodegraders (A) Gating strategy and representative flow cytometry plots obtained from the screening after transfection or not of different biodegrader plasmids into HeLa S3/H2B-GFP-ALFA-KRAS^G12V^_166_. SSC, side scatter; FSC, forward scatter; MFI, median fluorescence intensity. (B) Quantification of the GFP MFI by flow cytometry of HeLa S3/H2B-GFP-ALFA-KRAS^G12V^_166_ cells transfected with the indicated biodegrader plasmids. The intensity of GFP fluorescence in the mCherry transfected cells is analyzed by flow cytometry and compared to the GFP MFI of the negative control. Data represented in B are mean ± standard deviation (SD) of three independent biological experiments. Ctl, control. Statistical significance was determined by one-way ANOVA followed by Dunnett post-hoc test (ns, not significant; ∗∗∗∗*p* < 0.0001).

20. Plate 70,000 HeLa S3 / H2B-GFP-ALFA-KRAS^G12V^_166_ sorted cells per well of a 12-well plate with 1 mL of supplemented DMEM.
  a. 24 h after, transfect 0.8 μg of plasmid DNA of interest (coding for the biodegraders) with 1.6 μL of JetPRIME reagent.
  b. Change the medium in each transfected well with fresh supplemented DMEM 4 h after transfection.
21. 48 h later, prepare the cells for the flow cytometry-based screening (Troubleshooting 4).
  a. Aspirate the medium.
  b. Wash each well with 500 μL of PBS.
  c. Add 200 μL of trypsin-EDTA solution in each well to detach the cells.
  d. Add 300 μL of supplemented DMEM in each well to neutralize the trypsin.
  e. Mix well the cells by pipetting up and down.
  f. Transfer the cells into a 5 mL round-bottom tube.
  g. Add 2 mL of PBS and centrifuge at 800 × *g* for 5 min at 22°C.
  h. Remove the supernatant and add 300 μL of PBS-4% FBS.
22. Proceed with the flow cytometer analysis (see an example of gating Figure 4A).
  a. Select the cells population using the side and forward scatters’ area (SSC-A and FSC-A, respectively) channels that inform on the granularity and size of the cells, respectively.
  b. Remove the doublets by gating the cells on FSC-H (Height) and FSC-A channels.
  c. Among the single cells, select the mCherry positive cells (FSC-A, mCherry-A channels).
  d. Measure the GFP fluorescence intensity (median) in the mCherry positive population (FSC-A, GFP-A channels). Troubleshootings 5 and 6.

**CRITICAL:** Include a non-relevant biodegrader (negative control), i.e. that does not bind to the GFP-POI and therefore that does not degrade it. This control will serve as reference for the quantification of the flow cytometry data (see Figure 4B). If available, add a positive biodegrader, i.e. a biodegrader known to degrade the GFP-POI such as the anti-GFP nanobody GFP4^14^ in fusion with the VHL E3 ligase that was shown to deplete numerous GFP-POI.^15^^,^^16^ Also, include a non-transfected condition for gating purpose.

***Note:*** The screening could be scaled-up by seeding and then transfecting cells in a 96-well plate.

23. Transfect the positive hits to confirm the results of the screening by exactly repeating the steps 20–22.
24. Confirm that the degradation of the biodegrader hits is proteasome dependent. Repeat steps 20–22 with the following modifications.
  a. Transfect the plasmid DNA of each biodegrader hit in duplicate following the procedure described in step 20a.
  b. When changing the medium in the two transfected wells of a hit (step 20b.), add fresh complete DMEM with DMSO (solvent of the epoxomicin) in one well and 0.8 μM of epoxomicin (final concentration), a proteasome inhibitor, in the other well.
  c. Repeat that step for each hit.
  d. 20 h later, proceed as in steps 21a to 22d.

***Alternatives:*** Any other proteasome inhibitors can be used for the step 24 such as MG132 or Bortezomib.

## Expected outcomes

This protocol will enable the identification of new E3 ligases that function in a biodegrader setting, i.e., fused to an intracellular protein binder. We obtained around 50% of positive hits from the screening we previously performed,^1^ with a decrease of GFP median fluorescence intensity (MFI) ranging from ≈ 25 to 90% depending on the hits. However, this number will depend on multiple parameters such as the number of tested E3 ligases, the truncation of the E3 ligase used, their positioning on the sdAb, … After that screening, it will be possible to characterize the versatility of the identified E3 ligase(s)-based biodegrader(s) in different cell lines, using different subcellular localization for the POI or different intracellular binders. Importantly, it is strongly advised to test the ability of the E3 ligase-based biodegrader to deplete an endogenous target in a cell line of interest. To do that, the biodegrader should be expressed in relevant cell lines by a lentivector, with inducible promoter if required, as previously reported.^1^^,^^17^^,^^18^

## Quantification and statistical analysis

For the screening, we quantified the GFP median of fluorescence using flow cytometry. The MFI is analyzed in the mCherry transfected cells and the results are given as normalized GFP MFI in percentage: the MFI of the biodegrader is divided by the MFI of the negative control and multiplied by 100.

Because our screening included a small number of E3 ligases to test (7 in total, example of results in Figure 4B), we performed it three times. The subsequent statistical analysis of the screening data is done by using a one-way ANOVA followed by Dunnett post-hoc tests (all conditions are compared to the negative control). A biodegrader is considered as positive if its normalized GFP MFI is statistically decreased compared to the negative control. If a higher number of E3 ligases is screened and the screen is not repeated several times, therefore the statistical analysis to identify the positive hits should be adapted.

## Limitations

A limitation of this protocol is that the screening is performed on a chimeric POI that is exogenously expressed. A way to circumvent the overexpression issue could be to add the fluorescent protein directly on the POI using a gene editing method such as CRISPR-Cas9. Of note, the gene editing might be time consuming and difficult depending on the POI to tag.

The truncation of the E3 ligase is done to reduce the size of the E3 ligase but also to remove any unnecessary domain that could interfere with the expression level or subcellular localization of the E3 ligase. Nevertheless, the truncation might modulate the activity of the corresponding biodegrader. Therefore, it could be possible to employ full-length E3 ligases as reported elsewhere.^19^

Each biodegrader may have a different expression level in cells, which might affect their degradation efficiency and the screening outcomes. Depending on the throughput of the screening, it is advised to control this expression level by Western blot as we previously performed^1^ (exemplified in Figure 3).

Screening assays are meaningful only if confirmation steps are performed afterward. If the screening is performed once, then every positive hit should be retested using the same settings as in the screening (see step 23). Before starting any in-depth analysis of a new E3 ligase-based biodegrader, it is important to check that the decrease of fluorescence measured by flow cytometry is proteasome dependent as described in step 24.

Depending on the desired features of the E3 ligase (e.g., versatile or specific of a subcellular compartment), it will be mandatory to assess the newly discovered E3 ligase-based biodegrader in various cell lines expressing the same POI (e.g., H2B-GFP-ALFA-KRAS^G12V^_166_) or with different subcellular localizations.

Finally, a validation step will be required to determine whether the newly identified E3 ligase-based biodegrader enables the degradation of an endogenously expressed POI, in a relevant cell line as we previously performed.^1^^,^^17^^,^^18^

## Troubleshooting

### Problem 1

All cells are dead after the tri-transfection (step 3).

### Potential solution

Reduce the DNA quantity of lentiviral vector and accordingly the volume of JetPrime reagent (step 2).

### Problem 2

All cells are dying after transduction (step 13).

### Potential solution

Reduce the volume of virions-containing supernatant for the transduction (step 12).

Purify the suspension of virions by ultracentrifugation to remove any carryover cell contaminants, as previously described.^20^

### Problem 3

All cells are dying after antibiotic selection (steps 14–15).

### Potential solution

Reduce the concentration of antibiotic. A killing curve for each cell line and antibiotic used should be performed to determine the right concentration of antibiotic to employ during the selection.

### Problem 4

Cells die after transfection with the biodegrader plasmids (step 21).

### Potential solution

Increase the quantity of cells plated.

Decrease the amount of plasmid DNA to transfect (e.g., 0.4 μg of DNA and 0.8 μL of JetPrime reagent).

Perform the flow cytometry analysis after 24 h of transfection instead of 48 h (this might result in lower degradation efficacy).

### Problem 5

Cells do not express the biodegrader(s) (no red fluorescence detected by flow cytometry, step 22).

### Potential solution

Check the construct by sequencing.

Increase the amount of biodegrader plasmid DNA to transfect.

Use another ratio of JetPrime reagent or another transfection reagent.

### Problem 6

No positive hits after the cell-based screening (step 22).

### Potential solution

Verify that the POI can be degraded by defining a positive control before the screening.

Check that the stable cell line still has a homogenous expression of the GFP-tagged POI.

Check that the E3 ligase domain fused to the intracellular single domain antibody has been properly designed (i.e., truncation of the E3, position of the truncated E3 on the sdAb).

Increase the number of E3 ligases to screen.

Choose a narrow gate within the GFP positive population with a rather medium GFP intensity for the cell sorting. A too high intensity might affect the degradation efficiency of the biodegraders (too many targets to degrade).

## Resource availability

### Lead contact

Further information and requests for resources and reagents should be directed to and will be fulfilled by the lead contact, Nicolas Bery (nicolas.bery@inserm.fr).

### Technical contact

Technical questions on executing this protocol should be directed to and will be answered by the technical contact, Nicolas Bery (nicolas.bery@inserm.fr).

### Materials availability

This study did not generate new unique reagents.

### Data and code availability

No data or code were generated in this study.

## Acknowledgments

This work was financially supported by SANOFI (collaboration agreement SANOFI/Inserm Transfert SA/Université Toulouse III Paul Sabatier). M.S. is supported by a fellowship of the Ligue Nationale contre le Cancer. M.C. was supported by a fellowship of the Fondation FONROGA. A.C. was supported by a CIFRE fellowship (N°2020/1123) funded in part by the National Association of Research and Technology (ANRT) on behalf of the French Ministry of Education and Research and in part by Sanofi. N.B. was supported by fellowships from La Fondation de France (N°00097692, 00112961, 00119146).

## Author contributions

Conceptualization, M.S., M.C., A.C., and N.B.; methodology, M.S., M.C., A.C., and N.B.; writing, all authors; supervision and funding acquisition, K.D., P.C., and N.B.

## Declaration of interests

A.C. and K.D. are Sanofi employees and may hold shares and/or stock options in the company.
